# The Impact of Heavy Rainfall Variability on Fertilizer Application Rates: Evidence from Maize Farmers in China

**DOI:** 10.3390/ijerph192315906

**Published:** 2022-11-29

**Authors:** Jiangying Guo, Jiwei Chen

**Affiliations:** College of Economics and Management, Nanjing Agricultural University, Nanjing 210095, China

**Keywords:** heavy rainfall variability, fertilizer application rates, risk aversion, China

## Abstract

Global warming increases the intensity and frequency of extreme weather events, which is harmful to agricultural production. Given that the overuse of fertilizer has been found to be a significant contributor to global warming, it is crucial to analyze the factors affecting farmers’ fertilizer use and find appropriate fertilizer reduction measures. Agriculture is inherently risky, but previous studies have ignored the risk factors related to heavy rainfall variability, including fertilizer losses and the possible yield risks, which may lead to income risk. Using the 1995–2018 National Rural Fixed Observation Point Survey Data, this study examines the impact of heavy rainfall variability on maize farmers’ fertilizer application rates, aiming to understand farmers’ fertilization behavior in response to weather shocks. The results show that heavy rainfall variability significantly increases farmers’ fertilizer application rates on maize. Furthermore, we find that heavy rainfall variability has greater effects on fertilizer use in hills and mountainous areas and areas with good irrigation conditions or high economic levels. When examining the potential channels underlying the estimated effects, we find that yield fluctuations are a channel through which heavy rainfall variability affects farmers’ fertilizer use on maize. The above results indicate that farmers view applying extra fertilizer as a risk reducing activity in response to rainfall shocks, which helps to prevent low yield and income. Strengthening agricultural infrastructure construction according to local conditions and promoting fertilizer reduction technologies and products to reduce yield risk caused by heavy rainfall can help alleviate the problem of high fertilizer application rates by Chinese farmers.

## 1. Introduction

According to the IPCC Sixth Assessment Report (AR6), global surface temperature has increased faster since 1970 than in any other 50-year period over a least the last 2000 years, leading to an increase in the intensity and frequency of extreme weather events, which have severe harmful effects on agricultural production. In the agricultural sector, the overuse of fertilizer has been found to be a significant contributor to global warming [1,2,3]. In addition, the overuse of fertilizer has caused a series of soil problems, such as non-point source pollution and soil nutrient imbalance, hindering the sustainable development of agriculture [4]. Therefore, it is necessary to examine the factors affecting farmers’ fertilization behavior and then design corresponding policies to reduce fertilizer use.

In China, farmers’ fertilizer application rates are far higher than the internationally accepted safety limit of 225 kg/ha [5,6]. The Chinese government has introduced a series of measures to reduce farmers’ fertilizer application rates. For example, in 2015, the Chinese Ministry of Agriculture introduced the “Action to Achieve Zero Growth of Chemical Fertilizer Use by 2020”. Since then, the “No. 1 Document” of the central government has repeatedly emphasized the issue of fertilizer reduction for six consecutive years. However, the National Farm Product Cost-benefit Survey Data show that fertilizer application rates of different crops continued to increase slowly until 2020 (see Table A1 in the Appendix A), which means that considerable reductions in fertilizer use still need to be made.

Many studies have examined the factors influencing farmers’ fertilization behavior in China. First, production incentive policies and fertilizer price control policies formulated by the Chinese government have resulted in a high ratio of grain to fertilizer prices, which leads to deviations in the marginal output from the actual price of fertilizer [7,8,9,10]. Second, the scarcity of arable land, migration of the agricultural labor force, and rise of labor costs have led to the substitution of fertilizer for the above factors [11,12]. Third, characteristics of farmers, such as small-scale operations [10,13], high-volume fertilization habits [14], and risk aversion [15], have led to the high fertilizer application rates. Fourth, information asymmetry, such as fertilizer product updates that exceed farmers’ ability to assimilate the information [16], fertilizer nutrient contents on packaging that are higher than the actual contents [17], and asymmetry in soil quality information [18], is another reason for the high fertilizer application rates by Chinese farmers.

However, studies on the determinants of fertilizer used by Chinese farmers have ignored the risk factors related to heavy rainfall, including fertilizer losses and the possible yield risk [10,14,15,16]. Heavy rainfall in China is mainly concentrated in the summer when crops are growing and their demand for fertilizer is high. From the time it is applied into soil to its uptake by the crop, fertilizer can easily be washed away by heavy rainfall, resulting in leakage and losses, which greatly reduces fertilizer use efficiency [19]. Meanwhile, heavy rainfall may cause waterlogging in farmland (affecting the respiration of crop roots) and a high-humidity environment (more likely to lead to the occurrence of pests and diseases), thereby causing a crop yield reduction [20]. Increasing fertilizer use can supplement the loss of fertilizer and reduce the adverse impact of heavy rainfall on crop yield. Furthermore, heavy rainfall patterns are uncertain, and the interannual variability in heavy rainfall may cause an interannual change in yield. Therefore, farmers cannot accurately predict rainfall levels for the year when applying fertilizer, and farmers in areas with high rainfall variability may apply more fertilizer to avoid yield risk (ensuring stable income). 

Due to the synergistic relationship between soil nutrient uptake and water effectiveness, previous studies have mainly focused on examining the effect of rainfall on fertilizer use and found that the relationship is uncertain, depending on the level of economic development or agricultural infrastructure in different regions [21,22,23,24,25,26]. Only a few studies have examined farmers’ fertilization behavior under rainfall variability and found that rainfall abundance in the previous year increases fertilizer application rates by increasing yield and relaxing the liquidity limits of farmers, while rainfall variability decreases fertilizer application rates because it increases the possibility of harvest failure [27,28,29]. 

However, the findings of these studies may not be applicable to China. First, these studies mainly focus on promoting farmers’ fertilizer application rates in semi-arid and rain-fed areas with high interannual rainfall variabilities, such as Ethiopia and Tanzania, where fertilizer application rates are low and economic conditions are poor (farmers face high liquidity constraints) [27,28,29]. Given the high fertilizer application rates in China, which is different from these countries, the Chinese government mainly focuses on reducing farmers’ fertilizer application rates while maintaining yield [5,14]. Second, previous studies do not distinguish between rainfall intensity, which is problematic because normal rainfall and heavy rainfall have different effects on crop production (normal rainfall can improve yield, while heavy rainfall is unfavorable to production). This study defines daily rainfall exceeding 25 mm as heavy rainfall and examines the effect of heavy rainfall variability on farmers’ fertilizer use in China and its possible mechanisms.

Given that, as food for humans and livestock, maize has the largest sown area in China (requiring a large amount of fertilizer), its fertilizer application rates rank second among the three major crops (rice, wheat, and maize) (see the China agricultural products cost-benefit compilation of information). Reducing fertilizer use for maize plays an important role in decreasing fertilizer application rates in China. Meanwhile, as maize grows in summer with its higher probability of heavy rainfall, heavy rainfall may have a greater effect on maize farmers’ fertilizer use. Therefore, this study uses maize farmers to examine the impact of heavy rainfall variability on fertilizer application rates.

This study makes the following contributions. First, this is the first study to empirically examine the impact of rainfall variability on maize farmers’ fertilizer application rates in China, which is conducive to providing a supplementary explanation for the high fertilizer application rates by Chinese farmers. Second, this study adopts heavy rainfall variability as the proxy variable of income risk and examines its impact on farmers’ fertilizer application rates, which has important reference value for the design of fertilizer reduction policies. Third, in light of similar existing literature, this study uses panel data (which are the largest longitudinal household survey in rural China, covering more than 350 villages every year) and daily rainfall data (which can accurately identify rainfall intensity) to explicitly examine farmers’ responses to rainfall variability. 

The remainder of this study is organized as follows. Section 2 discusses the theoretical framework. Section 3 describes the data and methodology used. Section 4 presents the empirical results and robustness checks. Section 5 presents further discussion on potential channels. Conclusions are presented in Section 6.

## 2. Theoretical Analysis and Hypothesis Proposal

### 2.1. Risk Characteristics of Fertilizer

Previous studies have generally defined the input that leads to greater or lower yield variance as “risk-increasing” or “risk-reducing” inputs, respectively [30,31]. Many studies have found that fertilizer can increase yield variance, which is a “risk-increasing” input, and risk-averse farmers should theoretically apply less fertilizer under risk [30,32,33]. However, they prefer to apply more fertilizer in reality [15,34].

To explain the divergence between the reality and theory, two issues need to be clarified. First, do farmers apply more fertilizer to reduce variation or loss? Farmers do not avoid upward fluctuations in yield and income, and mainly avoid downward fluctuations. If applying more fertilizer can reduce the possibility of yield reduction, farmers have an incentive to apply more fertilizer. Second, is risk the cause or consequence of applying more fertilizer? If yield and income variability are thought to be caused mainly by the high fertilizer application rates, then risk-averse farmers will theoretically apply less fertilizer. However, owing to the uncertainty of agricultural production, external risk should be a more important factor affecting farmers’ fertilization behavior. Moreover, previous studies have found that farmers seem to have a different understanding of fertilizer from economists. Farmers generally argue that fertilizer does not increase risk, but instead they use more fertilizer as a means to reduce risk [34,35,36,37,38]. Therefore, under risk conditions, farmers are inclined to apply more fertilizer to prevent low yield and income [32,36,39].

### 2.2. Heavy Rainfall Variability and Risk-Averse Farmers’ Fertilizer Use

Agricultural production and management are risky, and farmers are risk-averse with the goal of utility maximization (pursuing the trade-off between profit maximization and income stability). Therefore, the strict marginal analysis framework, which assumes that farmers pursue profit maximization, is no longer suitable for analyzing farmers’ fertilization behavior, and the equilibrium condition of fertilization is no longer that the price of fertilizer is equal to the value of marginal products. Given that farmers generally consider that applying more fertilizer can mitigate the negative effect of uncertain production conditions, they tend to prevent unacceptable decreases in income by applying more fertilizer to maximize utility when there are production risks.

Therefore, applying more fertilizer will break the original equilibrium between the original marginal benefits and marginal costs, leading to the right shift of the equilibrium point, which seems to be “economically undesirable”. However, applying more fertilizer increases the income expected value and its distribution, ensuring that farmers’ income is not too low even in years of bad weather, thus maximizing their utility. Therefore, under risk conditions, farmers will weigh the costs and benefits of applying more fertilizer. The equilibrium point of fertilizer use remains at the second stage of production, where the yield has not yet reached the maximum and the marginal yield is greater than zero.

**Figure 1:**
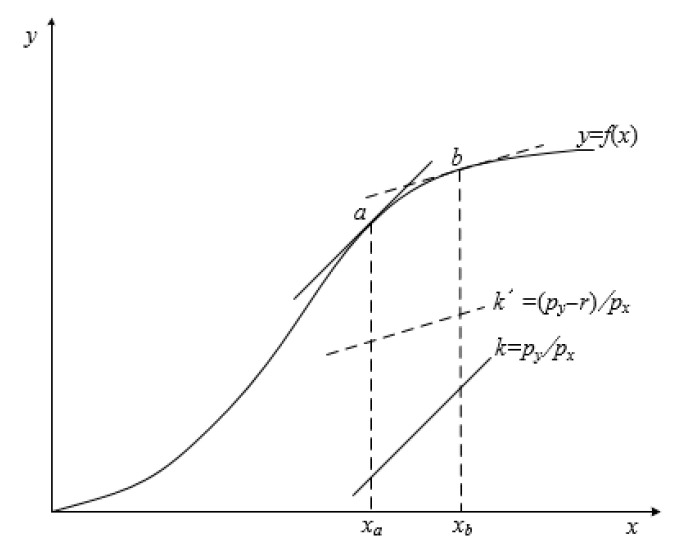
Utility maximization and farmers’ fertilizer use.

Figure 1 shows the relationship between utility maximization and farmers’ fertilizer use. Specifically, it is known that farmers pursue utility maximization:

(1) $$\max.{\;U=p}_{y}y-p_{x}x+U_{r}$$

where *x* denotes fertilizer application rates, *y* denotes yield (*y* ≤ *f*(*x*), *f*(*x*) denotes the production function under certain technology), and *U_r_* denotes the utility of income stabilization from additional fertilizer (*U_r_* = *rx* and production risk factor *r* ≥ 0). Under certain risk, *U_r_* increases with fertilizer application rates in the second production stage.

When there is no production risk (*r* = 0), the slope of the iso-utility line (the iso-profit line) is *k* = *p_x_*/*p_y_*, the equilibrium point of fertilizer use is *a*, and the fertilizer application rates are *x_a_*. When there is production risk (*r* > 0), the slope of the iso-utility line is *k’* = (*p_x_* − *r*)/*p_y_*, the equilibrium point of fertilizer use is *b*, and the fertilizer application rates are *x_b_*. *x_b_* > *x_a_* indicates that farmers apply more fertilizer to avoid production risk.

The characteristics of agricultural production and fertilizer have led farmers to apply more fertilizer under risk conditions. First, because there are many uncertainties in agricultural production, and fertilizer is a yield-enhancing input that can compensate for the loss of yield caused by an excess or shortage of other factors to a certain extent. Second, the process of fertilizer uptake by crops is special because fertilizer application and crop nutrient uptake are not synchronized. Nutrient uptake by crops is continuous, while fertilizer is applied once or in stages, with most fertilizers needing a period after application before they are absorbed. During this period, adverse weather may cause fertilizer losses, resulting in yield losses. When the above situations cause yield losses, farmers can increase crop yield and reduce yield risk by applying more fertilizer. 

**Figure 2:**
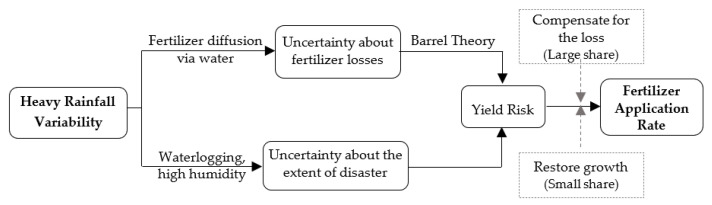
Heavy rainfall variability and farmers’ fertilizer use.

Therefore, it is necessary to apply additional fertilizer to restore plant growth and compensate for fertilizer losses owing to heavy rainfall. Heavy rainfall causes not only waterlogging in farmland, which affects crop metabolism, but also high humidity conditions, leading to the spread of diseases and multiplication of insect pests, thus causing crop yield reductions. If heavy rainfall occurs before the middle term of crop growth, foliar fertilizer can be sprayed to quickly restore crop growth by improving the photosynthesis of crop leaves. However, foliar fertilizer accounts for a small proportion of the total fertilizer use. The amount of fertilizer required to compensate for the fertilizer losses caused by heavy rainfall is relatively large. Meanwhile, heavy rainfall in China mainly occurs in summer when crops require more fertilizer and the concentration of fertilizer in the soil is relatively high. The scouring and leaching effect of heavy rainfall on fertilizer is relatively large, which causes the leakage and runoff of fertilizer. According to the barrel theory, crop yield depends on the input of the “shortest board”. If additional fertilizer is not applied after heavy rainfall, serious crop losses may occur owing to fertilizer runoff. Figure 2 shows the impact of heavy rainfall variability on farmers’ fertilizer use.

Meanwhile, rainfall is random and heavy rainfall has significant interannual variations. Given that farmers cannot accurately predict rainfall levels for the year when making fertilizer decisions (fertilization is an ex ante action), they can only make rough estimates based on rainfall levels in previous years. Therefore, in areas with large rainfall levels and variability in previous years, farmers may apply more fertilizer to ensure that yield and income are not too low. In addition, poor detectability of soil nutrient contents, nutrient losses, and crop uptakes results in incomplete information on fertilizer use efficiency, exacerbating farmers’ uncertainty about fertilizer application rates. Farmers tend to apply more fertilizer to ensure sufficient nutrients for crop growth.

It should be noted that fertilization is mainly an ex ante decision rather than an ex post disaster relief, and the occurrence of risk is random. Avoiding risk requires more fertilizer to be systematically applied annually rather than just adding fertilizer after risks occur. The additional fertilizer applied each year can be seen as a fixed cost to achieve the expected goal. Moreover, although farmers seem to increase fertilizer use to avoid yield risk, they actually avoid income risk (applying more fertilizer to ensure that the probability and extent of yield reduction are not too large and income is not too low).

Thus, we propose the following two hypotheses:

(1) There is a positive relationship between heavy rainfall variability and farmers’ fertilizer application rates on maize.
(2) Yield fluctuations are a channel through which heavy rainfall variability affects farmers’ fertilizer application rates on maize.

## 3. Data and Methodology

### 3.1. Data Source

This study uses the National Rural Fixed Observation Point Survey Data in China, which have been operated since 1986, covering more than 350 administrative villages and more than 20,000 farmers in 31 provinces in China, excluding Hong Kong, Macao, and Taiwan. It is the largest longitudinal household survey in rural China, providing detailed information on the basic characteristics of villages, farmers, and family members as well as their production and living conditions.

We obtain data on rainfall intensity by matching the National Rural Fixed Observation Point Survey Data with farming season data and meteorological data (from the National Meteorological Information Center of the China Meteorological Administration). The farming season data represent a dataset of crop growth and farmland soil moisture decadal values in China, providing records on crop growth and development, through 778 stations across China since 1991, which can be used to calculate the development time of maize at each site and accurately identify the fertilization concentration period. Meanwhile, the meteorological data come from the daily dataset of surface climate data in China (V3.0), which contains 699 benchmark and basic weather station data collected since 1951. The daily dataset can accurately identify rainfall intensity during the fertilization concentration period of maize (the period from sowing to heading when the concentration of fertilizer in soil is relatively high and heavy rainfall has a larger effect on fertilizer use efficiency) and examine its effect on farmers’ fertilizer application rates.

### 3.2. Research Design

We adopt the following linear regression model to investigate the effect of heavy rainfall variability on farmers’ fertilizer application rates on maize:

(2) $${\mathrm{Ferter}}_{vt}=\beta_{0}+\beta_{1}RF_{vt}+\beta_{2}R_{vt}+\gamma X_{vt}+Z_{v}{+T}_{t}+Z_{v}\times T_{t}+\varepsilon_{vt}$$

where *Ferter_vt_* denotes farmers’ fertilizer application rates on maize of village *v* in period *t*, and *RF_vt_* denotes the interannual fluctuation of rainfall during the concentrated fertilization period in the previous eight years of village *v* in period *t*. *R_vt_* represents the annual average rainfall during the concentrated fertilization period in the previous eight years of village *v* in period *t*. *X_vt_* represents a set of village characteristics that may affect farmers’ fertilizer application rates, including fertilizer/grain price ratio, average cultivated land area, irrigation conditions, terrain, and village economic level. *Z_v_* represents province fixed effects, controlling for time-invariant province-level characteristics, such as the concept of fertilization of local residents. *T_t_* denotes year fixed effects, controlling for omitted variables that change over time at the national level, such as food security levels and fertilizer reduction policies. Z_v_*T_t_ represents the interaction terms between province and year dummies, controlling for time-variant province-level characteristics that may affect fertilizer use, such as inflation, provincial economic level, and the local government’s emphasis on food production. Finally, *ɛ_vt_* is a random error term.

Furthermore, we adopt the following model to investigate the effect of heavy rainfall variability on yield fluctuations and test whether yield fluctuations are a channel for heavy rainfall variability to affect farmers’ fertilizer use on maize:

(3) $$YF_{vt}=\beta_{0}+\beta_{1}RF_{vt}+\beta_{2}R_{vt}+\gamma X_{vt}+Z_{v}{+T}_{t}+Z_{v}\times T_{t}+\varepsilon_{vt}$$

where *YF_vt_* denotes the interannual fluctuation of maize yield in the previous eight years of village *v* in period *t*, and *X_vt_* represents a set of village characteristics that may affect yield fluctuations, including lagged grain price fluctuations (given farmers’ reaction to grain prices and the lag of the response of agricultural product output to price change, we control for the lagged grain price fluctuations), fertilizer price fluctuations (given that fertilizer input is an important component of grain production cost and the change of fertilizer price may lead to yield fluctuations, we control for fertilizer price fluctuations), average cultivated land area, irrigation conditions, terrain, and village economic level. The other variables are the same as those in Equation (2).

### 3.3. Variable Construction

Following Ji et al. [14] and Gao et al. [16], we use the average amount of fertilizer applied per mu (1 mu = 0.0667 hectare) of maize at the village level to measure fertilizer application rates. Furthermore, we calculate the pure amount of fertilizer per mu of maize (the amount of nutrients, such as nitrogen, phosphorus, and potassium, contained in fertilizer) by matching the National Rural Fixed Observation Point Survey Data with the National Farm Product Cost-benefit Survey Data for robustness checks.

Fertilizer losses are mainly related to rainfall intensity and fertilizer concentration in the soil. Given the scouring effect of heavy rainfall on fertilizer, to more accurately identify the impact of heavy rain scouring on fertilizer losses, we combine the knowledge of soil science [40], and define the daily rainfall exceeding 25 mm as heavy rainfall. Meanwhile, regardless of the traditional fertilization method (apply fertilizer after seeding/planting and fertilize multiple times) or advanced fertilization method (fertilize at sowing time, “one cannon” fertilizer, and a combination of base fertilizer and topdressing fertilizer), maize fertilization is mainly concentrated on the sowing date (base fertilizer) and the maize trumpeting stage (top-dressing, about ten days before heading). Given that fertilizer losses caused by heavy rainfall increase with the nutrient concentration in soil, according to the growth and fertilization rules of maize combined with the residence time of fertilizer in soil, we define the period from sowing to heading when the concentration of fertilizer in soil is relatively high as the concentrated fertilization period of maize, and use rainfall intensity in the concentrated fertilization period to measure heavy rainfall.

To more accurately identify the impact of heavy rainfall on farmers’ fertilizer use, we divide the standard deviation of heavy rainfall into the variation coefficient of heavy rainfall and the mean value of heavy rainfall. Heavy rainfall refers to the total rainfall (daily rainfall exceeding 25 mm) during the concentrated fertilization period each year. Therefore, the mean value of heavy rainfall refers to the average value of village-level heavy rainfall in the previous eight years. The variation coefficient of heavy rainfall refers to the variation coefficient of village-level heavy rainfall in the previous eight years.

Owing to the advancement of agricultural technology, a significant time trend is observed in crop yield. We use the H-P filtering method to eliminate the time trend components [41] and retain the short-term fluctuation components for subsequent analysis. Specifically, yield fluctuations are calculated as follows:

First, we use the H-P filtering method to decompose the yield *Y_vt_* into fluctuating yield *a_vt_* and trend yield *u_vt_*. Then, we use the following formula to calculate the root mean square of all fluctuating yields *a_vt_* in the past *N* years:

(4) $$YF_{vt}=\sqrt{\frac{1}{N}\overset{N}{\underset{t=1}{\sum }}a_{vt}^{2}}$$

where *v* denotes village, *t* denotes year, *a_vt_* denotes the fluctuating yield, and *u_vt_* denotes the trend yield. Furthermore, we use the H-P filtering method to calculate lagged fertilizer price fluctuations and grain price fluctuations.

Farmers apply fertilizer mainly based on a cost-benefit comparison. According to the fertilization equilibrium conditions in the traditional analysis framework (Δ*F* = *P_grain_*/*P_fertilizer_*), the amount of fertilizer applied by farmers may increase with the fertilizer/grain price ratio. Therefore, the fertilizer/grain price ratio may be one of the reasons for the high fertilizer application rates by Chinese farmers. Meanwhile, since farmers are the price recipients of fertilizer and grain, the prices of fertilizer and grain faced by farmers in the same village should be the same. Therefore, we use the average price of fertilizer and grain of farmers in a village to represent the price of fertilizer and grain separately [16]. Given that farmers cannot predict the grain price when applying fertilizer and can only refer to the grain price in previous years, we use the ratio of grain price in the previous year and fertilizer price in the current year to measure the fertilizer/grain price ratio.

Table 1 reports the definitions and summary statistics of key variables. It should be noted that we use the 1995–2018 National Rural Fixed Observation Point Survey Data to calculate income fluctuations, price fluctuations, and yield fluctuations, and use the 2003–2018 National Rural Fixed Observation Point Survey Data to calculate other variables, such as fertilizer application rates and village characteristics. The National Rural Fixed Observation Point Survey Data usually contain one sample village in each city. Meanwhile, Table 1 shows that between-group standard deviations of most variables are higher than within-group standard deviations. Therefore, we control for province fixed effects and use the difference of heavy rainfall variability among different villages in the same province to investigate its impact on farmers’ fertilizer application rates.

Table 2 reports changes of fertilizer application rates for maize (the pure amount of fertilizer per mu of maize) in China. Overall, we found that fertilizer application rates for maize in China were slowly rising from 2003 to 2020. There are obvious differences in fertilizer application rates in different regions, which may be the result of the long-term effects of production and market conditions. Specifically, fertilizer application rates are the highest in Northwest China and slightly lower in East, North, and Northeast China. Fertilizer application rates in Central, Southwest, and South China are relatively stable, with little change from 2003 to 2020. The above results indicate that Chinese farmers have been applying high amounts of fertilizer for a long time, and fertilizer application rates have maintained a relatively high and stable level over the past 20 years.

**Figure 3:**
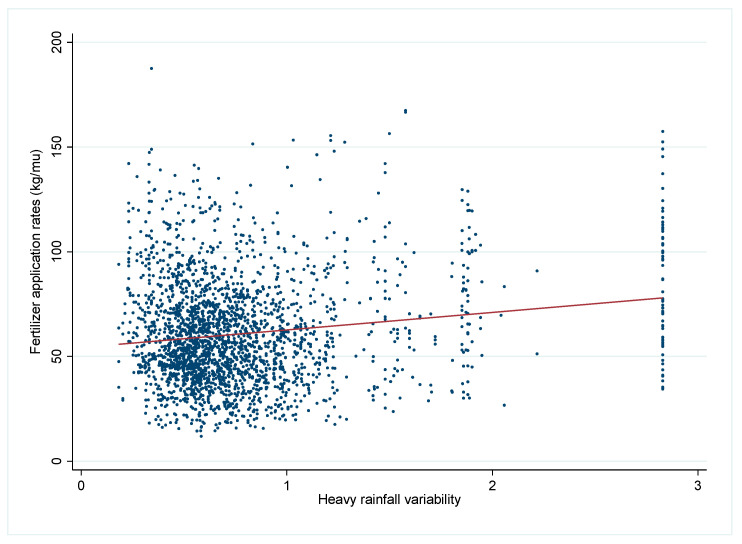
The relationship between heavy rainfall variability and fertilizer use.

Figure 3 reports the scatter plot and linear fit line between heavy rainfall variability and fertilizer application rates, showing that there is a positive correlation between heavy rainfall variability and farmers’ fertilizer application rates.

## 4. Empirical Results

### 4.1. Effect of Heavy Rainfall Variability on Fertilizer Application Rates

Table 3 reports the impact of heavy rainfall variability on farmers’ fertilizer application rates on maize. Column (1) controls for province and year fixed effects, and the results show that heavy rainfall variability has a positive significant impact on fertilizer application rates. Column (2) adds village characteristics, and the results remain similar. Column (3) further adds the interactions between province and year dummies, controlling for the time-variant province level characteristics. The results shows that heavy rainfall variability significantly increases fertilizer application rates. Specifically, farmers’ fertilizer application rates will increase by 1.7 kg for a 0.1 increase in historical heavy rainfall variability. Relative to the average fertilizer application rates of 60.84 kg/mu, farmers’ fertilizer application rates will increase by 13.4% (4.8 × 1.7/60.84) for each one standard deviation (0.48) increase in heavy rainfall variability, which means that heavy rainfall variability is one of the reasons for the high fertilizer application rates by Chinese farmers.

Meanwhile, we find that heavy rainfall significantly increases farmers’ fertilizer application rates. Farmers’ fertilizer application rates will increase by 0.5 kg for a 1 cm increase in historical heavy rainfall. The above results indicate that heavy rainfall increases possible fertilizer losses, and the interannual fluctuation of heavy rainfall leads to uncertainty in fertilizer losses. When farmers make fertilizer decisions, heavy rainfall variability increases the uncertainty of the available nutrient content in the soil (rainfall is random and fertilizer decisions are made in advance). Given that farmers do not know rainfall level of the year when applying fertilizer, they can only refer to the rainfall situation in previous years. If farmers anticipate a possible fertilizer shortage, they will apply more fertilizer to ensure yield, thus preventing the possibility of low income. Our findings are inconsistent with those of Alem et al. [27], Gebrehaweria & Stein [28], Dercon & Christiaensen [29], and Arslan et al. [42]. The possible reason is that these studies mainly focus on semi-arid and rain-fed areas (e.g., Ethiopia and Tanzania) with high interannual rainfall variability, where farmers face great uncertainty in production and high liquidity constraints. Heavy rainfall leads to farmers who lack capital and consumption smoothing mechanisms to reduce fertilizer use, thus preventing harvest failure. However, farmers’ liquidity constraints are lower and fertilizer prices are cheaper in China. Farmers in areas with large heavy rainfall variability tend to apply more fertilizer to ensure that the probability and extent of yield reduction is not too large and income is not too low. 

Furthermore, the fertilizer/grain price ratio has a significant positive effect on fertilizer application rates, indicating that the long-term control and direct subsidy policies implemented by the Chinese government on the fertilizer industry to ensure food security and protect the interests of farmers have resulted in high grain prices and low fertilizer prices, which is one of the reasons for the higher fertilizer application rates in China. This finding is in line with Ge & Zhou [7], Li et al. [8], and Wu et al. [10]. Irrigation conditions have a positive impact on fertilizer application rates. There are two reasons why irrigation conditions affect farmers’ fertilizer use. First, in areas with good irrigation conditions, farmers have higher production boundaries and maize needs more fertilizer. Second, as the main fertilization method in China, flood irrigation also leads to the high fertilizer application rates by farmers to compensate for the yield losses caused by heavy rainfall. However, the effects of irrigation conditions on farmers’ fertilizer application rates are statistically insignificant. This may be because irrigation makes water and fertilizer more coordinated, and better absorption of nutrients by crops reduces fertilizer application rates to a certain extent, thus offsetting the negative impact of irrigation on fertilizer application rates. Villages with a higher economic level have high fertilizer application rates, which may be owing to higher levels of non-farm employment and lower grain self-sufficiency rates in these areas. Therefore, to improve the self-sufficiency rate of grain, farmers in these areas tend to apply more fertilizer to increase crop yield and income.

### 4.2. Robustness Checks

Next, we test the robustness of the main results. First, heavy rainfall is determined by the local climate, and not by the behavior of farmers, indicating that heavy rainfall is a strictly exogenous variable. Second, we control for a set of village characteristics, province fixed effects, year fixed effects, and the interaction terms between province and year dummies (eliminating the endogeneity problem caused by omitted variables). Therefore, we argue that there is no obvious endogeneity problem in Equation (2). In addition, we also conduct a series of robustness checks (see Table 4).

Given that different kinds of fertilizers contain different nutrients, we use the average amount of fertilizer and may ignore the differences in the crop fertilization structure. Therefore, we calculate the pure amount of fertilizer per mu of maize by matching the National Rural Fixed Observation Point Survey Data (including the average fertilizer cost per mu of maize) with the cost-benefit compilation of information (including the average fertilizer cost per mu of maize and the pure amount of fertilizer in each province) for the robustness check. The results in column (1) are similar to those in Table 3. 

In the baseline regression, we use the data on rainfall intensity from the previous eight years to calculate the interannual fluctuation of heavy rainfall and the annual average rainfall, which may be biased owing to the length of the measurement period. We further use a shorter time period of five years and a longer time period of ten years for robustness checks. The results in columns (2) and (3) change marginally. Heavy rainfall variability has significant positive effects on fertilizer application rates.

### 4.3. Heterogeneous Effects

First, we investigate whether heavy rainfall variability has a heterogeneous effect on maize farmers’ fertilizer application rates by terrain. The results show that heavy rainfall variability significantly increases farmers’ fertilizer application rates in area consisting of plains, hills, and mountains, and the estimates are greater for farmers in hills and mountainous areas (see the first two columns of Table 5). The possible reason for this is that, owing to the relatively poor growing conditions and imperfect infrastructure in hills and mountainous areas compared to plain areas, maize yield may be less affected by natural risks, which exacerbates the uncertainty of farmers on yield. Therefore, the effect of heavy rainfall variability on farmers’ fertilizer use is greater in hills and mountainous areas. 

Second, farmers often apply fertilizer at the same time as irrigation, and flood irrigation is still the main method in China, which may cause fertilizer run-off due to heavy rainfall scouring. Therefore, in areas with good irrigation conditions, heavy rainfall will increase fertilizer run-off and yield uncertainty, and heavy rainfall variability may have a greater impact on farmers’ fertilizer application rates. We divide villages into two groups according to the mean value of irrigation conditions. The results in columns (3) and (4) of Table 5 show that there is obvious heterogeneity in the impact of heavy rainfall variability on maize farmers’ fertilizer application rates, and heavy rainfall variability has a greater effect on fertilizer use in areas with good irrigation conditions. 

Third, we further divide villages into two groups according to village economic levels to investigate the heterogeneous impact of heavy rainfall variability on fertilizer application rates. The results show that heavy rainfall variability has a greater effect on fertilizer application rates in areas with good economic conditions (see the last two columns of Table 5). The possible reason is that farmers have small, cultivated land areas and low commodity rates in areas with good economic conditions, and their maize yield may not meet household consumption. Therefore, under uncertain conditions, farmers in these areas tend to increase fertilizer use to achieve self-sufficiency in food.

### 4.4. Potential Mechanisms

In this section, we examine the effect of heavy rainfall variability on yield fluctuations to test whether yield fluctuations are a channel for heavy rainfall variability to affect farmers’ fertilizer application rates on maize. The results in Table 6 show that heavy rainfall variability significantly increases yield fluctuations. Specifically, yield fluctuations will increase by 1.29 kg for a 0.1 increase in historical heavy rainfall variability. Relative to the average yield fluctuations of 60.3 kg/mu, yield fluctuations will increase by 10.3% (4.8 × 1.29/60.3) for each one standard deviation (0.48) increase in heavy rainfall variability, indicating that the probability and extent of yield reduction increases with heavy rainfall variability and farmers apply more fertilizer to reduce yield and income risks. This finding is in line with Urban et al. [43], Pattanayak & Kumar [44], Powell & Reinhard [45], and Murari et al. [46]. These studies have found that extreme weather events, such as extreme high temperatures and extreme precipitation events can significantly decrease crop yield and are regarded as the principle immediate threat to global crop production. 

However, the observed yield fluctuations are the result of long-term excessive fertilizer use by farmers under the influence of the natural, social, and economic production environments in the region. To reduce yield reductions caused by fertilizer losses owing to heavy rainfall, farmers may choose to apply more fertilizer, which means that what we observed is yield fluctuations after farmers have applied more fertilizer. It should be noted that owing to the limited ability to remedy and restore plant growth after disasters, even if farmers apply more fertilizer, they cannot completely compensate for the negative impact of heavy rainfall variability on crop yield. Although the observed yield fluctuations occur after farmers applied more fertilizer, we found that heavy rainfall variability has significant positive effects on yield fluctuations. Therefore, we may underestimate the impact of heavy rainfall variability on yield fluctuations.

## 5. Further Discussion

In addition to examining the impact of heavy rainfall variability on farmers’ fertilizer application rates on maize, it is important to understand farmers’ perceptions of fertilizer use efficiency and their response measures when facing heavy rainfall. In this section, we use the Jiangsu Province Rural Fixed Observation Point Survey Data, collected by Nanjing Agricultural University in eight counties (Donghai, Sihong, Dafeng, Baoying, Danyang, Lishui, Haimen, and Kunshan) in January 2022 to examine farmers’ perceptions of fertilizer use efficiency and their response measures to heavy rainfall, testing the mechanism of the impact of heavy rainfall variability on maize farmers’ fertilizer application rates. Table 7 reports the distribution of maize and rice farmers.

When farmers were asked “Would a 10% reduction in current fertilizer application rates have an impact on yield?”, Table 8 shows that 72% of maize farmers and 84% of rice farmers argued that this scenario would have an impact on yield. When fertilizer application rates are assumed to decrease by 20%, the number of maize and rice farmers who argue that yield will be affected reaches 92% and 100%, respectively, indicating that most farmers argue that existing fertilizer application rates are appropriate and reducing fertilizer application rates will adversely affect yield. Perhaps because of this fertilization perception, it is difficult for farmers to voluntarily reduce fertilizer use without a change in technology, which is the main reason for the poor outcomes of fertilizer reduction work. Therefore, farmers’ perceptions of the impact of fertilizer reduction on crop yield may be a reason for the high fertilizer application rates by Chinese farmers.

When farmers were asked, “When applying base fertilizer, if 30% of the fertilizer is estimated to be lost due to waterlogging within half a month, will you apply more fertilizer?”, Table 9 shows that 56% of maize farmers and 44% of rice farmers argued that they would increase base fertilizer (adding fertilizer before disasters represents a preventive fertilization practice, while adding fertilizer after disasters represents a remedial fertilization practice). When farmers were asked, “If waterlogging occurs and may reduce the yield by 30%, will you apply topdressing?”, the results show that 70% of maize farmers and 76% of rice farmers argued that they would apply additional fertilizer (remedial fertilization practice). Furthermore, we found that 45 maize farmers and 17 rice farmers answered that they would increase fertilizer use before and after disasters, indicating that more than half of the farmers would take both preventive and remedial actions. The above results suggest that farmers apply more fertilizer mainly to prevent and remedy the adverse effects of natural risk (heavy rainfall variability) on crop yield. Farmers view applying more fertilizer as a means to avoid risks, that is, by applying more fertilizer to ensure that crops can absorb sufficient nutrients and reduce the likelihood of yield reductions, which is in line with Rajsic et al. [32], Babcock [36], Yang et al. [37], and Stuart et al. [38]. 

## 6. Conclusions and Policy Implications

This study uses farmers’ responses to changes in rainfall levels to examine whether heavy rainfall variability is a reason for the high fertilizer application rates of Chinese farmers. Using the 2003–2018 National Rural Fixed Observation Point Survey Data, we found that farmers’ fertilizer application rates on maize increase with heavy rainfall variability, indicating that heavy rainfall variability is a reason for the high fertilizer application rates by Chinese farmers. Farmers’ fertilizer application rates will increase by 1.7 kg for a 0.1 increase in heavy rainfall variability. Furthermore, we investigated the heterogeneous effects of heavy rainfall variability on fertilizer use, and found that heavy rainfall variability has greater effects on fertilizer application rates in hills and mountainous areas and areas with good irrigation and economic conditions. Finally, we examined the potential channels behind the estimated effects and found that yield fluctuations are a channel through which heavy rainfall variability affects fertilizer application rates. The above results indicate that heavy rainfall variability increases the risk or uncertainty associated with income and yield and farmers apply more fertilizer to prevent yield and income risk.

The findings of this study indicate that yield uncertainty caused by heavy rainfall variability leads to the high fertilizer application rates by Chinese farmers. When making fertilizer decisions, farmers not only rely on the traditional marginal revenue analysis framework, but also consider the impact of natural risk. To reduce farmers’ fertilizer application rates, the Chinese government can promote corresponding fertilizer reduction technologies (deep loosening and deep application) and products (slow- and controlled-release fertilizers) through financial subsidies (e.g., agricultural machinery purchases and service subsidies, policy and financial support to enterprises to reduce the price of environmentally friendly fertilizers, and subsidies for farmers to purchase slow- and controlled-release fertilizers) and demonstration households, to reduce fertilizer losses caused by heavy rainfall and improve fertilizer use efficiency. Furthermore, the Chinese government can reduce fertilizer losses and yield reduction caused by heavy rainfall by strengthening agricultural infrastructure construction (e.g., promoting farmland reconstruction projects and implementing farmland protection measures according to local conditions).

## Figures and Tables

**Table 1:** Definitions and summary statistics of key variables.

Variables	Definition	Mean	Standard Deviation		
			Over-All	Between-Group	Within-Group
Fertilizer application rates	Fertilizer application rates per unit area at village-level (kg/mu)	60.84	25.22	22.15	12.94
Heavy rainfall variability	Interannual fluctuation of heavy rainfall during the fertilization concentration period in the previous 8 years	0.78	0.48	0.49	0.20
Heavy rainfall	Annual average heavy rainfall during the fertilization concentration period in the previous 8 years (cm)	12.24	7.51	7.29	2.42
Fertilizer/grain price ratio	Ratio of grain price in the previous year and fertilizer price in the current year	0.84	0.34	0.29	0.18
Irrigation conditions	Average proportion of irrigated land in farmland operated by farmers	0.70	0.33	0.31	0.09
Terrain	Plain	0.46	0.50	0.49	0.11
	Hills or mountainous areas	0.54	0.50	0.49	0.11
Village economic level in its county	Below medium	0.18	0.38	0.32	0.21
	Medium	0.45	0.50	0.40	0.30
	Above medium	0.37	0.48	0.40	0.27
Average cultivated land area	Average cultivated land per household at village-level (mu/household)	10.74	9.71	9.14	2.94
Yield fluctuations	Interannual fluctuation of maize yield in the previous 8 years (kg/mu)	60.30	33.52	29.86	17.43
Lagged grain price fluctuations	Lagged interannual fluctuation of grain price in the previous 8 years (yuan)	0.22	0.09	0.08	0.05
Fertilizer price fluctuations	Interannual fluctuation of fertilizer price in the previous 8 years (yuan)	0.30	0.15	0.13	0.09

Note: Data come from the 2003–2018 National Rural Fixed Observation Point Survey.

**Table 2:** Changes of fertilizer application rates for maize in China (unit: kg/mu).

Year	Nationwide	North China	Northeast China	East China	Central China	South China	Southwest China	Northwest China
2003	21.17	16.70	18.37	20.13	21.60	20.70	23.20	25.28
2004	20.67	18.43	15.56	19.63	18.96	16.79	24.33	25.15
2005	20.02	18.36	16.17	20.30	19.16	21.27	20.24	23.87
2006	20.93	19.66	19.18	21.11	19.59	23.02	20.33	23.79
2007	21.93	20.39	20.18	22.64	20.53	23.36	20.39	25.73
2008	21.01	19.55	19.85	21.11	20.66	23.69	20.62	22.82
2009	22.32	19.16	21.51	22.60	21.02	21.88	22.58	25.58
2010	23.75	20.74	22.57	22.90	23.08	25.32	22.77	28.44
2011	23.50	21.03	23.06	23.21	22.49	23.56	21.06	28.85
2012	23.80	20.64	24.09	24.78	23.03	22.65	21.37	28.34
2013	24.06	21.71	24.49	25.74	22.53	24.18	21.62	27.42
2014	25.20	23.21	25.44	27.27	24.08	25.16	21.54	29.20
2015	25.10	23.38	25.32	26.52	23.06	20.78	22.21	30.17
2016	25.47	24.28	25.45	26.73	22.62	22.76	22.35	30.68
2017	25.59	24.38	25.64	26.24	22.24	23.84	22.61	31.04
2018	25.23	24.83	25.73	25.53	22.41	22.46	21.82	30.44
2019	25.07	24.43	24.95	24.46	22.05	19.69	22.42	31.60
2020	26.10	24.52	25.57	26.37	22.84	22.85	23.12	32.92

Note: Data come from the China agricultural products cost-benefit compilation of information.

**Table 3:** Effect of heavy rainfall variability on fertilizer application rates.

	(1)	(2)	(3)
Heavy rainfall variability	11.533 ***	12.430 ***	17.296 ***
	(2.082)	(1.861)	(2.237)
Heavy rainfall	0.298 ***	0.373 ***	0.498 ***
	(0.096)	(0.085)	(0.095)
Fertilizer/grain price ratio		42.209 ***	41.152 ***
		(1.779)	(1.905)
Irrigation conditions		1.695	1.568
		(1.490)	(1.583)
Average cultivated land area		0.143	−0.090
		(0.119)	(0.192)
Terrain (Plain = 1)			
Hills or mountainous areas		−1.044	−1.132
		(1.035)	(1.067)
Village economic level (Below medium = 1)			
Medium		−0.578	−0.783
		(1.192)	(1.252)
Above medium		4.191 ***	4.276 ***
		(1.228)	(1.270)
Year fixed effects	YES	YES	YES
Province fixed effects	YES	YES	YES
Province * year fixed effects	NO	NO	YES
R^2^	0.261	0.442	0.488
RMSE	22	19	18
Observations	2482	2482	2482

Note: Data come from the 2003–2018 National Rural Fixed Observation Point Survey. Robust standard errors are reported in parentheses. * *p* < 0.10; ** *p* < 0.05; *** *p* < 0.01.

**Table 4:** Robustness checks using alternative specifications.

	(1)	(2)	(3)
Heavy rainfall variability	9.145 ***	12.050 ***	16.325 ***
	(1.097)	(2.038)	(2.095)
Heavy rainfall	0.348 ***	0.329 ***	0.516 ***
	(0.050)	(0.087)	(0.096)
Control variables	YES	YES	YES
Year fixed effects	YES	YES	YES
Province fixed effects	YES	YES	YES
Province * year fixed effects	YES	YES	YES
R^2^	0.663	0.477	0.490
RMSE	15	18	18
Observations	2473	2456	2494

Note: Data come from the 2003–2018 National Rural Fixed Observation Point Survey. All regressions control for fertilizer/grain price ratio, average cultivated land area, irrigation conditions, terrain, and village economic level. Robust standard errors are reported in parentheses. * *p* < 0.10; ** *p* < 0.05; *** *p* < 0.01.

**Table 5:** Heterogeneous effects of heavy rainfall variability on fertilizer application rates.

	Terrain		Irrigation Conditions		Village Economic Level	
	Plain	Hills and Mountainous	Poor	Good	Poor	Good
Heavy rainfall variability	7.554 **	8.341 ***	10.711 ***	19.800 ***	15.742 ***	22.550 ***
	(3.828)	(2.719)	(3.100)	(3.155)	(2.992)	(3.704)
Heavy rainfall	0.096	0.244 *	0.655 ***	0.491 ***	0.488 ***	0.725 ***
	(0.165)	(0.145)	(0.161)	(0.146)	(0.120)	(0.198)
Control variables	YES	YES	YES	YES	YES	YES
Year fixed effects	YES	YES	YES	YES	YES	YES
Province fixed effects	YES	YES	YES	YES	YES	YES
Province * year fixed effects	YES	YES	YES	YES	YES	YES
R^2^	0.648	0.562	0.606	0.548	0.502	0.640
Observations	1153	1329	991	1491	1558	924

Note: Data come from the 2003–2018 National Rural Fixed Observation Point Survey. All regressions control for fertilizer/grain price ratio, average cultivated land area, irrigation conditions, terrain, and village economic level. Robust standard errors are reported in parentheses. * *p* < 0.10; ** *p* < 0.05; *** *p* < 0.01.

**Table 6:** Effects of heavy rainfall variability on yield fluctuations.

	(1)	(2)	(3)
Heavy rainfall variability	9.715 ***	9.445 ***	12.901 ***
	(2.575)	(2.550)	(3.012)
Heavy rainfall	−0.163	−0.212	−0.098
	(0.165)	(0.165)	(0.184)
Lagged grain price fluctuations		24.908 **	15.711
		(9.773)	(9.993)
Fertilizer price fluctuations		5.577 *	9.950 ***
		(3.021)	(3.535)
Irrigation conditions		−3.206	−2.689
		(2.814)	(2.958)
Average cultivated land area		−0.307	1.749 ***
		(0.247)	(0.275)
Terrain (Plain = 1)			
Hills or mountainous areas		−5.334 ***	−5.034 ***
		(1.649)	(1.721)
Village economic level (Below medium = 1)			
Medium		−8.226 ***	−8.602 ***
		(1.956)	(2.065)
Above medium		−10.642 ***	−10.424 ***
		(2.105)	(2.211)
Year fixed effects	YES	YES	YES
Province fixed effects	YES	YES	YES
Province * year fixed effects	NO	NO	YES
R^2^	0.225	0.241	0.294
RMSE	30	29	28
Observations	2482	2482	2482

Note: Data come from the 2003–2018 National Rural Fixed Observation Point Survey. Robust standard errors are reported in parentheses. * *p* < 0.10; ** *p* < 0.05; *** *p* < 0.01.

**Table 7:** The distribution of maize and rice farmers.

City	County	Maize Farmers	Rice Farmers
Lianyungang	Donghai	19	0
Suqian	Sihong	20	4
Yancheng	Dafeng	18	3
Yangzhou	Baoying	0	13
Zhenjiang	Danyang	2	0
Nanjing	Lishui	4	5
Nantong	Haimen	25	0

Note: Data come from the Rural Fixed Observation Point Survey in Jiangsu (2022). There are no suitable farmers because all cultivated land in surveyed villages is transferred in Kunshan County, Suzhou City.

**Table 8:** Farmers’ perception of the impact of fertilizer reduction on yield.

Question	Crop Type	Yes (%)	How Much Will Yield Decrease? (%)				
			Below 10	10–20	20–35	35–50	Above 50
Would a 10% reduction in current fertilizer use have an impact on yield?	Maize	72	46	33	11	3	7
	Rice	84	67	24	0	0	9
Would a 20% reduction in current fertilizer use have an impact on yield?	Maize	92	25	29	19	17	10
	Rice	100	20	44	20	12	4

Note: Data come from the Rural Fixed Observation Point Survey in Jiangsu (2022).

**Table 9:** Waterlogging disasters and farmers’ fertilizer application behavior.

Question	Crop Type	Yes (%)	How Much More Fertilizer Should Be Applied? (%)				
			Below 10	10–20	20–35	35–50	Above 50
When applying base fertilizer, if 30% of the fertilizer is estimated to be lost due to waterlogging within half a month, will you apply more fertilizer?	Maize	56	31	26	25	12	6
	Rice	44	33	34	11	16	6
If waterlogging occurs and may reduce the yield by 30%, will you apply topdressing?	Maize	70	21	19	28	19	13
	Rice	76	37	31	11	5	16

Note: Data come from the Rural Fixed Observation Point Survey in Jiangsu (2022).

## Data Availability

Not applicable.

## Appendix A

**Table A1:** Total fertilizer application rates and fertilizer application rates (the pure amount of fertilizer per mu) of different crops in China.

Year	Total Fertilizer Application Rates (10,000 Tons)	Maize (kg/mu)	Wheat (kg/mu)	Japonica Rice (kg/mu)	Middle Indica Rice (kg/mu)	Late Indica Rice (kg/mu)
2000	4146.41	22.50	22.00	23.10	19.40	19.80
2005	4766.22	18.39	21.59	22.58	19.74	20.38
2010	5561.68	22.51	25.15	24.39	18.93	21.09
2015	6022.60	24.30	27.05	24.06	20.06	22.39
2016	5984.41	24.82	27.35	23.87	20.50	23.14
2017	5859.41	24.88	27.67	24.61	20.30	22.76
2018	5653.42	24.78	27.41	24.30	20.53	22.70
2019	5403.59	24.36	28.13	24.96	21.37	23.12
2020	5250.65	24.97	28.33	26.02	21.08	22.92

Note: Total fertilizer application rates come from China Statistics Yearbook, and fertilizer application rates of different crops come from the China Agricultural Products Cost-benefit Compilation of Information.

## References

[1] Kahrl F., Li Y., Su Y., Tennigkeit T., Wilkes A., Xu J. (2010). Greenhouse gas emissions from nitrogen fertilizer use in China. Environ. Sci. Policy.

[2] Ma Q., Zheng S., Deng P. (2022). Impact of internet use on farmers’ organic fertilizer application behavior under the climate change context: The role of social network. Land.

[3] Menegat S., Ledo A., Tirado R. (2022). Greenhouse gas emissions from global production and use of nitrogen synthetic fertilisers in agriculture. Sci. Rep..

[4] Chen X., Pu M., Zhong Y. (2022). Evaluating China food’s fertilizer reduction and efficiency initiative using a double stochastic meta-frontier method. Int. J. Environ. Res. Public Health.

[5] Fang P., Abler D., Lin G., Sher A., Quan Q. (2021). Substituting organic fertilizer for chemical fertilizer: Evidence from apple growers in China. Land.

[6] Guo Y., Wang J. (2021). Spatiotemporal changes of chemical fertilizer application and its environmental risks in China from 2000 to 2019. Int. J. Environ. Res. Public Health.

[7] Ge J., Zhou S. (2012). Does factor market distortions stimulate the agricultural non-point source pollution? A case study of fertilizer. Issues Agric. Econ..

[8] Li Y., Zhang W., Ma L., Huang G., Oenema O., Zhang F., Dou Z. (2013). An analysis of China’s fertilizer policies: Impacts on the industry, food security, and the environment. J. Environ. Qual..

[9] Smith L.E., Siciliano G. (2015). A comprehensive review of constraints to improved management of fertilizers in China and mitigation of diffuse water pollution from agriculture. Agric. Ecosyst. Environ..

[10] Wu Y., Xi X., Tang X., Luo D., Gu B., Lam S.K., Vitousek P.M., Chen D. (2018). Policy distortions, farm size, and the overuse of agricultural chemicals in China. Proc. Nat. Acad. Sci. USA.

[11] Yan Z., Liu P., Li Y., Ma L., Alva A., Dou Z., Chen Q., Zhang F. (2013). Phosphorus in China’s intensive vegetable production systems: Overfertilization, soil enrichment, and environmental implications. J. Environ. Qual..

[12] Wu W., Liu Y. (2017). The impact of non-agricultural income on input structure of agricultural factors under the background of rural labour migration. Chin. J. Popul. Sci..

[13] Huan M., Zhan S. (2022). Agricultural production services, farm size and chemical fertilizer use in China’s maize production. Land.

[14] Gao J., Peng C., Shi Q. (2019). Study on the high chemical fertilizer consumption and fertilization behavior of small rural household in China: Discovery from 1995-2016 National Fixed Point Survey Data. J. Manag. World.

[15] Qiu H., Luan H., Li J., Wang Y. (2014). The impact of risk aversion on farmers’ behavior of excessive fertilizer application. Chin. Rural. Econ..

[16] Ji Y., Zhang H., Lu W., Liu H. (2016). Differentiation, incomplete information, and farmers’ excessive fertilizer application. J. Agrotech. Econ..

[17] Khor L.Y., Zeller M. (2014). Inaccurate fertilizer content and its effect on the estimation of production functions. China Econ. Rev..

[18] Xu X., He P., Zhang J., Pampolino M.F., Johnston A.M., Zhou W. (2017). Spatial variation of attainable yield and fertilizer requirements for maize at the regional scale in China. Field Crops Res..

[19] Liu J., Zuo Q., Zhai L., Luo C., Liu H., Wang H., Liu S., Zou G., Ren T. (2016). Phosphorus losses via surface runoff in rice-wheat cropping systems as impacted by rainfall regimes and fertilizer applications. J. Integr. Agric..

[20] Zhou L., Zhou S. (2012). Post-disaster adaptability to extreme weather events. China Popul. Resour. Environ..

[21] Kaliba A.R., Verkuijl H., Mwangi W. (2000). Factors affecting adoption of improved maize seeds and use of inorganic fertilizer for maize production in the intermediate and lowland zones of Tanzania. J. Agric. Appl. Econ..

[22] Mohanam T.C. (2002). The Determinants of Fertilizer Consumption & Its Growth.

[23] Nyssen J., Biruk B., Tesfamariam Z., Frankl A., Demissie B., Ghebreyohannes T., Meaza H., Poesen J., Van Eetvelde V., Zenebe A. (2017). Geographical determinants of inorganic fertiliser sales and of resale prices in north Ethiopia. Agric. Ecosyst. Environ..

[24] Haider H., Smale M., Theriault V. (2018). Intensification and intrahousehold decisions: Fertilizer adoption in Burkina Faso. World Dev..

[25] Heisse C., Morimoto R. (2019). Climate Change, Chemical Fertilisers, and Sustainable Development: Panel Evidence from Tanzanian Maize Farmers.

[26] Bora K. (2022). Rainfall shocks and fertilizer use: A district level study of India. Environ. Dev. Sustain..

[27] Alem Y., Bezabih M., Kassie M., Zikhali P. (2010). Does fertilizer use respond to rainfall variability? Panel data evidence from Ethiopia. Agric. Econ..

[28] Gebrehaweria G., Stein H. (2011). Does irrigation enhance and food deficits discourage fertilizer adoption in a risky environment? Evidence from Tigray, Ethiopia. J. Dev. Agric. Econ..

[29] Dercon S., Christiaensen L. (2011). Consumption risk, technology adoption and poverty traps: Evidence from Ethiopia. J. Dev. Econ..

[30] Roosen J., Hennessy D.A. (2003). Tests for the role of risk aversion on input use. Am. J. Agric. Econ..

[31] Antle J.M. (2010). Asymmetry, partial moments, and production risk. Am. J. Agric. Econ..

[32] Rajsic P., Weersink A., Gandorfer M. (2009). Risk and nitrogen application levels. Can. J. Agric. Econ..

[33] Monjardino M., McBeath T.M., Brennan L., Llewellyn R.S. (2013). Are farmers in low-rainfall cropping regions under-fertilising with nitrogen? A risk analysis. Agric. Syst..

[34] Paulson N.D., Babcock B.A. (2010). Readdressing the fertilizer problem. J. Agric. Resour. Econ..

[35] SriRamaratnam S., Bessler D.A., Rister M.E., Matocha J.E., Novak J. (1987). Fertilization under uncertainty: An analysis based on producer yield expectations. Am. J. Agric. Econ..

[36] Babcock B.A. (1992). The effects of uncertainty on optimal nitrogen applications. Appl. Econ. Perspect. P.

[37] Yang X., Fang S., Lant C.L., Luo X., Zheng Z. (2012). Overfertilization in the economically developed and ecologically critical Lake Tai region, China. Hum. Ecol..

[38] Stuart D., Schewe R.L., McDermott M. (2014). Reducing nitrogen fertilizer application as a climate change mitigation strategy: Understanding farmer decision-making and potential barriers to change in the US. Land Use Policy.

[39] Sheriff G. (2005). Efficient waste? Why farmers over-apply nutrients and the implications for policy design. Appl. Econ. Perspect. P.

[40] Liu X., Lu K., Li P., Xu G., Cheng S., Bai L., Wei F. (2018). Research and simulation of soil water infiltration on slope under different rainfall conditions. J. Arid. Land Resour. Environ..

[41] Hodrick R.J., Prescott E.C. (1997). Postwar US business cycles: An empirical investigation. J. Money Credit. Bank..

[42] Arslan A., Belotti F., Lipper L. (2017). Smallholder productivity and weather shocks: Adoption and impact of widely promoted agricultural practices in Tanzania. Food Policy.

[43] Urban D., Roberts M.J., Schlenker W., Lobell D.B. (2012). Projected temperature changes indicate significant increase in interannual variability of US maize yields. Clim. Chang..

[44] Pattanayak A., Kumar K.K. (2014). Weather sensitivity of rice yield: Evidence from India. Clim. Chang. Econ..

[45] Powell J.P., Reinhard S. (2016). Measuring the effects of extreme weather events on yields. Weather. Clim. Extrem..

[46] Murari K.K., Sandeep M., Jayaraman T., Madhura S. (2018). Extreme temperatures and crop yields in Karnataka, India. Rev. Agrar. Stud..

